# Alterations in innate immune defense distinguish first-episode schizophrenia patients from healthy controls

**DOI:** 10.3389/fpsyt.2022.1024299

**Published:** 2022-10-13

**Authors:** Song Chen, Mengzhuang Gou, Wenjin Chen, Meihong Xiu, Hongzhen Fan, Yunlong Tan, Li Tian

**Affiliations:** ^1^Peking University HuiLongGuan Clinical Medical School, Beijing HuiLongGuan Hospital, Beijing, China; ^2^Department of Physiology, Faculty of Medicine, Institute of Biomedicine and Translational Medicine, University of Tartu, Tartu, Estonia

**Keywords:** first-episode schizophrenia (FES), endotoxin, high mobility group box 1 protein (HMGB1), complement component 4, biomarker

## Abstract

Innate immune components involved in host defense have been implicated in schizophrenia (SCZ). However, studies exploring their clinical utility in SCZ diagnosis are limited. The main purpose of this study was to evaluate whether circulating endotoxin, high mobility group box 1 protein (HMGB1) and complement component 4 (C4) could act as peripheral biomarkers to distinguish first-episode schizophrenia (FES, *n* = 42) patients from healthy controls (HCs, *n* = 35) in associations with psychopathological symptoms and cognitive dysfunctions. Also, their changes after 8-week antipsychotic treatment were investigated. The Positive and Negative Syndrome Scale (PANSS), Psychotic Symptom Rating Scale (PSYRATS), and MATRICS Consensus Cognitive Battery (MCCB) were administered. Receiver operating characteristic (ROC) curves were conducted to evaluate the diagnostic effectiveness of the three biological indicators. Compared to HCs, levels of endotoxin, HMGB1, and C4 were remarkably increased in FES patients after controlling for age, gender, body mass index (BMI) and education years, and the combination of the three biomarkers demonstrated desirable diagnostic performance (AUC = 0.933). Moreover, the endotoxin level was positively correlated with the severity of auditory hallucinations. After 8 weeks of treatment, HMGB1 was decreased significantly in patients but still higher than that in HCs, whereas endotoxin and C4 did not change statistically. The baseline levels of endotoxin, HMGB1, and C4, as well as their changes were not associated with changes in any PANSS subscale score and total score. Our preliminary results suggest that a composite peripheral biomarker of endotoxin, HMGB1, and C4 may have accessory diagnostic value to distinguish SCZ patients from HCs. Additionally, endotoxin might be implicated in the pathogenesis of auditory hallucinations.

## Introduction

Although the exact etiology of schizophrenia (SCZ) is still largely unknown, accumulating evidence has demonstrated that immune dysfunctions, especially innate immunity as the first line of host defense, are intimately implicated in the pathophysiology of SCZ. The complex interactions between the immune system and brain may contribute to a diverse range of changes in cognition, mood and behavior in psychiatric disorders and has received considerable attention in recent years (1, 2). Accumulated evidence has demonstrated that SCZ is accompanied by an ongoing chronic low-grade inflammation as indicated by simultaneous increase of both pro-inflammatory (IL-1, IL-6, and TNF-α) and anti-inflammatory cytokines (IL-10), as well as complement activation (1).

Pattern recognition receptors (PRRs) and their associated ligands, as well as the complement system are essential components of the innate immunity. PRRs, such as toll-like receptors (TLRs) and receptors for advanced glycation end products (RAGE), not only detect the components of exogenous pathogens (i.e., pathogen-associated molecular patterns or PAMPs), such as Gram-negative bacterial endotoxin (i.e., lipopolysaccharide, LPS), but also are capable of recognizing endogenous molecules released by dying cells or damaged tissues (i.e., damage-associated molecular patterns or DAMPs) (3). High mobility group box 1 protein (HMGB1), a DNA binding protein, is released from damaged or necrotic cells to the extracellular matrix, then acts as a pro-inflammatory cytokine trigging immune-inflammatory responses primarily via binding RAGE and several TLR family members (i.e., TLR2, TLR4, and TLR9) (3–5) and is required in endotoxin tolerance (6). By activating TLRs and RAGE signaling pathways, these molecules initiate the activation of immune and inflammatory responses (3), which need to be tightly regulated so that pathogens can be cleared while avoiding inflammatory overactivation. Notably, as PAMPs/DAMPs and complement components are essential for innate immune signaling, the importance of not only their individual functions but also their interplay in inflammatory processes have been realized recently (7). For instance, complement components are key regulators of PRR-mediated clearance of PAMPs and DAMPs in host organic innate immune responses (7, 8).

Endotoxin has been implied in SCZ due to its harmful effect on the brain and behavior, as demonstrated in neurodevelopmental conditions such as maternal immune activation (MIA) (9–11). On the other hand, a population-based cohort study with linkage of Danish national registers also indicated that infection was more likely to increase the risk of SCZ in susceptible individuals, whereas bacterial infection was associated with the highest risk (12). HMGB1 can disrupt the blood brain barrier (BBB), activate microglia and induce neurotoxic effects as a late mediator of inflammation (13, 14), and it has been implicated in a variety of neurological diseases such as ischemic stroke, epilepsy, multiple sclerosis, Alzheimer’s disease, and Parkinson’s disease, etc. (13, 15, 16). Nevertheless, there have been few studies examining endotoxin or HMGB1 in SCZ patients (17–21), especially in first-episode individuals.

Additionally, a growing body of evidence shows that the complement pathway appears to be more active in SCZ (22), especially complement component 4 (C4) due to its complex genetic variation leading to increased neuronal complement deposition and synapse elimination, and the development of psychotic symptoms (23, 24). C4 overexpression by MIA in late gestation in animal models has been proven to induce hypoconnectivity in the prefrontal cortex (PFC) circuitry and SCZ-like deficits (25, 26). However, serological studies have yielded mixed results regarding dysregulation of C4 in patients with SCZ including first-episode schizophrenia (FES) or psychosis (FEP) (27–30). Anyhow, given the importance of C4 in SCZ, studies focusing on early phenotype of SCZ and minimizing confounding factors would provide unique research value.

Our previous study showed that FES patients had more dampened instead of enhanced monocytic TLR4 response to LPS as compared to the HCs (31). We speculated that it might be associated with endotoxin- or HMGB1-induced tolerance, in other words, the FES patients might have higher levels of TLR4 ligands in their peripheral blood. Additionally, endotoxin, HMGB1, and C4 may holistically affect SCZ patients, yet studies exploring them collectively to understand their joint potential clinical utility are extremely limited. Therefore, the primary purpose of this study was to evaluate whether these molecules could act as peripheral biomarkers individually or jointly to distinguish FES patients from HCs, alongside their relationships with psychopathological symptoms and cognitive functions. Moreover, we endeavored to look whether their levels would change after 8-week antipsychotic treatment, and their correlations with treatment efficacy.

## Materials and methods

### Subjects

Forty-two FES patients within 2 weeks of cumulative psychotropic drugs exposure and 35 healthy volunteers, who all were Han Chinese people, were recruited from the Beijing HuiLongGuan Hospital and local community, respectively. The disease duration of all the patients was less than 2 years (16.1 ± 5.4 months), i.e., they were in an early stage of the illness. These samples were previously utilized and have been described in detail in our previous immune-psychiatry study (31), except one healthy subject in which had not plasma left. After 8 weeks of treatment, only 20 patients left blood samples and completed assessment of the Positive and Negative Syndrome Scale (PANSS). All blood samples were obtained in the morning after overnight fasting. This research adhered to the Helsinki Declaration and was approved by the Medical Ethical Committee of Beijing HuiLongGuan Hospital.

### Measurement of plasma endotoxin, high mobility group box 1 protein, and C4 concentration

Plasma levels of endotoxin, HMGB1, and C4 were determined at baseline and at 8 weeks of follow-up. Four (4) ml blood samples were collected in EDTAK_2_ tubes at the time of our previous research (31), which were stored at −80°C until analyses. Concentrations of the three substances were determined using commercially available enzyme-linked immunosorbent assay kits from Andy gene, Beijing, China: endotoxin (Human0939, sensitivity: 1.0 ng/L), HMGB1 (Human1775, sensitivity: 1.0 ng/L), and C4 (Human1584, sensitivity: 5.0 ng/L). All assays were conducted in blinded manner, and each sample was analyzed in duplicate.

### Assessment of psychopathological symptoms and cognitive functions

PANSS and Matrics Consensus Cognitive Battery (MCCB) were used to evaluate the psychopathological symptoms and cognition, respectively, as previously described (31). Furthermore, the Psychotic Symptom Rating Scale (PSYRATS) was administered to some FES patients at baseline (*n* = 30). PSYRATS, a clinician-administered and semi-structured interview, were developed by Haddock et al. to evaluate the dimensionality of psychotic experiences, which consists of auditory hallucinations rating scale (AHRS) including 11 items and delusions rating scale (DRS) including 6 items scoring from 0 to 4 points, with higher scores indicating greater severity of symptoms (32).

### Statistical methods

Data were analyzed using the IBM SPSS Statistics for Windows version 21.0. The Shapiro-Wilkinson test was used to examine data distribution. Group comparisons were performed using independent *t*-test or Mann-Whitney *U*-test for continuous variables and Chi-square test for categorical data. Analysis of covariance with age, gender, body mass index (BMI) and education years as covariates were applied to compare the levels of endotoxin, HMGB1, and C4 between FES patients and HCs, and then partial η^2^ was further calculated separately to determine the effect size of the group differences. A paired sample *t*-test was used to evaluate paired data.

Pearson and Spearman correlation analyses were applied as appropriate to investigate correlations of endotoxin, HMGB1, and C4 levels with clinical data at baseline. To explore the relationships between the three biological measures and clinical efficacy, we conducted partial correlation analyses controlling for baseline values.

The Receiver Operating Characteristic (ROC) curve analysis was performed to evaluate the diagnostic effectiveness of each baseline biological measure for differentiating FES patients from HCs. Then a combined model including endotoxin, HMGB1, and C4 jointly was constructed with multivariate logistic regression analysis, and its performance was compared with each substance alone by calculating areas under the ROC curves (AUC) with DeLong’s test. These analyses were performed using the pROC package in R 3.6.3. Plots were generated with ggplot2. Benjamini-Hochberg FDR (BH-FDR) was used for multiple testing correction. Two-tailed *p* < 0.05 was considered as statistically significant.

## Results

### Comparisons of demographic characteristics and plasma endotoxin, high mobility group box 1 protein, and C4 levels between first-episode schizophrenia and healthy controls

The demographic and clinical data of all participants at baseline are listed in Table 1. There were no statistically significant differences in age, gender, smoking status, BMI, and inflammatory marker high-sensitivity C-reactive protein (hs-CRP). However, the educational level was lower in FES patients than HC group (*p* < 0.001). As expected, FES patients showed impaired performance across all MCCB domains relative to normal controls (all *p* < 0.01; Table 1).

**TABLE 1:** The demographic and clinical characteristics of first-episode schizophrenia patients and healthy controls at baseline.

Variables	FES (*n* = 42)	HC (*n* = 35)	t/z/χ ^2^/*F*-value	*P-value*
Age (years)^a^	25.21 ± 6.20	26.20 ± 4.15	−0.802	0.425
Gender (male/female)^b^	18/24	20/15	1.559	0.212
Duration of illness (months)	16.1 ± 5.4	NA	NA	NA
Education (years)^c^	12.22 ± 2.76	14.34 ± 1.85	−3.895	2.176 × 10^–4^
Smoker/Non-smoker^b^	3/39	6/29	1.008	0.315
BMI (kg/m^2^)^c^	21.55 ± 3.25	21.76 ± 2.46	−0.464	0.644
hs-CRP (mg/L)^a^	1.79 ± 2.41	1.20 ± 2.00	0.881	0.378
Endotoxin (ng/L)^d^	339.11 ± 34.46	296.02 ± 36.97	25.357	3.644 × 10^–6^
HMGB1 (μg/L)^d^	30.69 ± 3.39	25.20 ± 3.56	48.857	1.388 × 10^–9^
C4 (μg/mL)^d^	484.41 ± 50.63	436.54 ± 50.98	16.926	1.058 × 10^–4^
MCCB composite score^e^	44.08 ± 10.73	60.82 ± 7.08	39.395	3.575 × 10^–8^
Processing speed^e^	45.34 ± 11.70	58.40 ± 9.58	13.961	3.845 × 10^–4^
Attention/vigilance^e^	40.23 ± 11.20	58.13 ± 7.24	43.057	9.668 × 10^–9^
Working memory^e^	44.50 ± 11.71	58.25 ± 6.67	23.192	8.729 × 10^–6^
Verbal learning^e^	44.33 ± 12.73	55.60 ± 10.00	12.609	0.001
Visual learning^e^	44.36 ± 10.25	56.83 ± 7.05	27.087	1.905 × 10^–6^
Reasoning/problem solving^e^	49.21 ± 11.46	60.84 ± 6.07	12.294	0.001
Social cognition^e^	44.77 ± 11.06	54.39 ± 12.78	11.493	0.001
PANSS total score	80.43 ± 12.51	NA	NA	NA
Positive score	22.18 ± 5.97	NA	NA	NA
Negative score	18.78 ± 6.53	NA	NA	NA
General psychopathology score	39.47 ± 7.37	NA	NA	NA
PSYRATS (FES *n* = 30)				
Auditory hallucinations scale	13.13 ± 14.65	NA	NA	NA
Delusions scale	18.78 ± 4.82	NA	NA	NA

Data are presented as mean ± SD. NA, Not Applicable.

^a^Mann–Whitney *U*-test.

^b^Chi-square test.

^c^Student’s *t*-test.

^d^Analysis of covariance with age, gender, BMI, and education years as covariates.

^e^Analysis of covariance with education years as covariate.

Analysis showed that concentrations of endotoxin (partial η^2^ = 0.269), HMGB1 (partial η^2^ = 0.415), and C4 (partial η^2^ = 0.197) were all significantly higher in FES patients as compared to the HCs after controlling for age, gender, BMI, and education years (all *p* < 0.001 and FDR corrected *p* < 0.001; Table 1), with the largest effect size for HMGB1.

### Diagnostic performance evaluation of baseline plasma endotoxin, high mobility group box 1 protein, and C4

**FIGURE 1:**
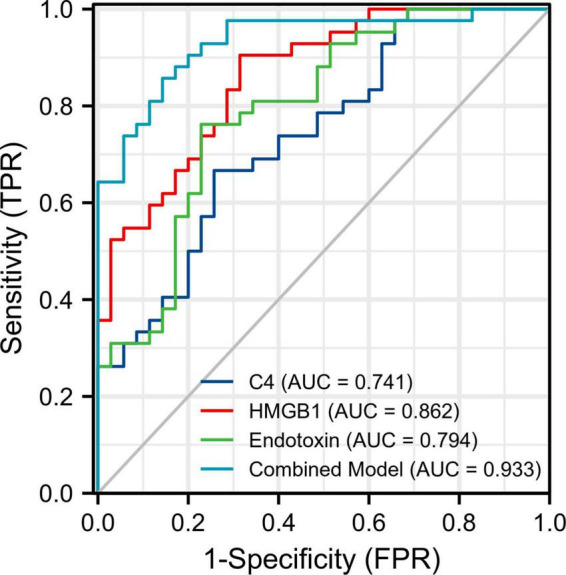
Receiver operating characteristic (ROC) curve analysis for discriminating first-episode schizophrenia patients from healthy controls. The areas under the ROC curves (AUC) of endotoxin, HMGB1, and C4 were calculated individually and in combination. FPR, false positive rate; TPR, true positive rate.

For a better utilization of the three peripheral biomarkers in the clinical diagnosis of SCZ, we evaluated their respective and combined diagnostic performance between the FES patients and the HCs. ROC analysis showed that the individual AUCs of endotoxin, HMGB1, and C4 ranged from 0.741 to 0.862 (95% CI ranged from 0.631 to 0.941), and the combination model of the three indicators significantly improved diagnostic power with an AUC of 0.933 (95% CI: 0.878–0.987; Figure 1 and Table 2). These results suggest that a composite biomarker had a better discrimination ability than any singular indicator (three comparisons, all FDR corrected *p* < 0.05; Table 2), with both sensitivity and specificity at 85.7% above the predicted probability cut-off value of 0.495 (Table 2; endotoxin: β = 0.033, *p* < 0.01; HMGB1: β = 0.411, *p* < 0.001; C4: β = 0.018, *p* < 0.05).

**TABLE 2:** ROC curve analysis for differentiating first-episode schizophrenia patients from healthy controls.

	AUC	95% CI	Cut-off	Sensitivity	Specificity	AUC comparisons	
						Statistics	FDR corrected *p*
Endotoxin (ng/L)	0.794	0.693–0.895	315.800	0.762	0.771	2.899^a^	<0.01
HMGB1 (μg/L)	0.862	0.783–0.941	26.325	0.905	0.686	2.219 ^a^	<0.05
C4 (μg/mL)	0.741	0.631–0.851	467.150	0.667	0.743	3.642 ^a^	<0.01
Combined model	0.933	0.878–0.987	0.495	0.857	0.857		

^a^Compared to combined model.

### Associations of baseline plasma endotoxin, high mobility group box 1 protein, and C4 with clinical assessments

No correlations of the baseline plasma endotoxin, HMGB1, and C4 were observed with age, gender, disease duration, BMI, smoking status, family history of psychiatric disorder, PANSS subscale scores, and total scores, as well as all cognitive dimensions evaluated by MCCB in FES group (all *p* > 0.05). Since our previous study had found that LPS stimulation resulted in a lower TLR4 expression on monocytes in FES patients as compared to HCs (31), and the blood samples in the present study were also from the same subjects, we further explored the associations of endotoxin, HMGB1, and C4 with monocytic TLR4 levels before and after LPS challenge. However, no significant relationships were observed (all *p* > 0.05). Additionally, the plasma endotoxin, HMGB1, and C4 were independent from each other in patient group (all *p* > 0.05). Results were the same in HC group.

**FIGURE 2:**
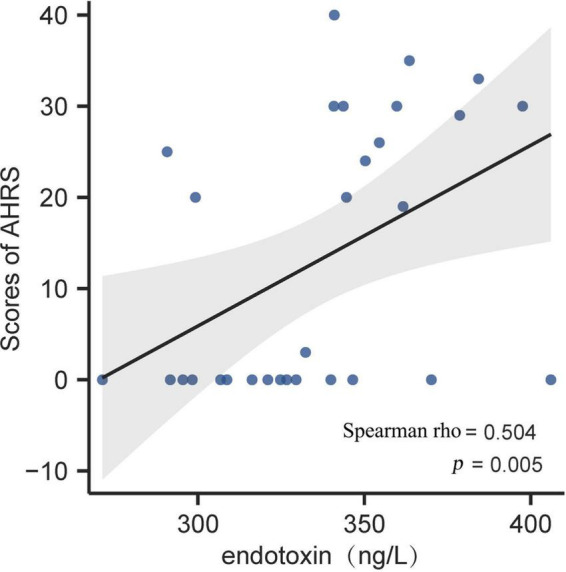
Correlation analysis between plasma endotoxin and AHRS score in first-episode schizophrenia (FES) patients at baseline. Scatter plots indicated that there was a significantly positive association of endotoxin concentration with auditory hallucinations in FES patients. The gray band represents 95% confidence interval. AHRS, auditory hallucinations rating scale.

Regarding the PSYRATS evaluation in the SCZ patients, we found a positive association between endotoxin concentration and baseline score of AHRS (Spearman rho = 0.504, *p* < 0.01; Figure 2). Beyond that, we observed no significant relationships neither between HMGB1, C4, and AHRS scores, nor their correlations with DRS scores (all *p* > 0.05).

### Changes in plasma endotoxin, high mobility group box 1 protein, and C4 after 8-week treatment as well as their associations with treatment efficacy

Eight-week follow up data were available for 20 patients with SCZ. There were significant improvements in PANSS positive scores, general psychopathology scores and total scores (all *p* < 0.001; Table 3) over the 8 weeks of treatment. As expected, negative symptoms did not improve in the short term (*p* > 0.05).

**TABLE 3:** Changes in plasma endotoxin, HMGB1, C4, and psychopathological symptoms of 20 patients after 8-week treatment.

Variables^a^	Baseline	After treatment	*t*-value	*P-value*
Endotoxin (ng/L)	335.23 ± 35.92	328.49 ± 43.73	0.683	0.503
HMGB1 (μg/L)	31.50 ± 3.01	29.26 ± 3.03	2.495	0.022
C4 (μg/mL)	490.42 ± 46.87	476.92 ± 50.96	0.799	0.434
PANSS total score	82.21 ± 11.69	55.95 ± 13.22	7.199	1.064 × 10^–6^
Positive score	22.50 ± 6.00	12.10 ± 3.71	6.926	1.330 × 10^–6^
Negative score	18.50 ± 6.19	16.00 ± 4.51	1.320	0.203
General psychopathology score	40.84 ± 7.58	27.68 ± 8.03	7.011	1.521 × 10^–6^

Data are presented as mean ± SD. ^a^Paired *t*-test.

There was a significant decrease in HMGB1 level after 8 weeks of treatment (*p* < 0.05; Table 3), but which was still remarkably higher than that in HCs (29.26 ± 3.03 vs. 25.20 ± 3.56, *t* = 4.283, *p* = 7.792 × 10^–5^). The levels of endotoxin and C4 did not change significantly (both *p* > 0.05; Table 3).

The levels of endotoxin, HMGB1, and C4 before and after treatment, as well as their changes were not associated with any PANSS subscale score, total score at 8 weeks and changes in PANSS scores by partial correlation controlling for baseline psychopathological symptoms (all *p* > 0.05).

## Discussion

Innate immune dysfunctions have been implied in the pathophysiology of SCZ. In this naturalistic longitudinal study, we selected endotoxin, HMGB1, and C4 as typical representatives of PAMPs, DAMPs, and complement system, respectively, and FES patients with minimal exposure to neuroleptic medications as subjects. There were three important findings. Firstly, circulating levels of endotoxin, HMGB1, and C4 were remarkably increased in FES patients as compared to HCs, and the combination of the three biomarkers showed desirable diagnostic performance in individuals during early stage of SCZ, with an impressive AUC of 0.933. Secondly, the baseline plasma endotoxin levels were found to be positively correlated with the severity of auditory hallucinations, indicating involvement of endotoxin in the pathophysiology of psychosis in FES. Thirdly, the level of plasma HMGB1, but not endotoxin or C4, was decreased significantly after 8 weeks of therapy, albeit still higher than that in HCs.

Immune-inflammatory response triggered by a variety of PAMPs and DAMPs, through PRRs, as well as complement pathway play critical roles in innate immune defense both individually and jointly (7). Normally, small amounts of endotoxin, released from dead Gram-negative bacteria primarily in the gut, are absorbed into the circulation through the intestinal mucosa. Noteworthily, many studies have demonstrated that SCZ is accompanied by increased intestinal permeability and impaired mucosal barrier function (leaky gut), giving rise to bacterial and endotoxin translocation to peripheral circulation (20, 33–35), which may explain the elevated endotoxin levels in FES patients observed in the current study. Endotoxin can destroy the BBB through a variety of mechanisms, and recruit immune mediators into the brain parenchyma, leading to neuroinflammation and disturbed neurotransmitter balance, with downstream consequences of sickness behavior (36, 37).

A recent study by Wang et al. revealed that serum levels of LPS and LPS-responded soluble CD14 were significantly higher in SCZ patients with aggression than in HCs and patients without any aggression, and they were not only positively correlated to proinflammation cytokines but also the severity of aggression (20). Moreover, peripheral anti-LPS IgA antibodies levels were also found to be associated with IL-6 elevation and recent suicide attempts in psychosis (18, 19). To our knowledge, ours is the first study to directly investigate plasma endotoxin level in FES, and we found a positive correlation between it and auditory hallucinations, which are a main positive symptom of SCZ. However, the underlying neurobiological mechanisms remain unclear. One possible explanation is that endotoxin-induced low-grade neuroinflammation could interfere with neurotransmitters, such as dopamine (38), and neurosteroids (39) in the cortical circuits. In an animal study, the corticocortical loop from the auditory association cortex to the orbitofrontal cortex, an underlying neural circuit involved in auditory hallucinations, was dramatically sensitive to dopamine and neurosteroids (40). In addition, it is also important to consider that auditory hallucination itself could evoke chronic psychological stress, which is a well-described gastrointestinal barrier disrupting factor (41), resulting in more endotoxin translocation to bloodstream.

Our result showed that HMGB1 concentration had the largest effect size for difference between the FES patients and HCs, as indicated by partial η^2^, among the three molecules we measured. A few studies have described the involvement of HMGB1 in psychosis, and our results are highly consistent with existing findings in different stages of SCZ, including patients with FES (21), patients in acute exacerbation phase and chronic patients (17, 42, 43), which all display that SCZ is characterized by increased levels of HMGB1 as compared with HCs. HMGB1 is released by two distinct pathways: passive release by damaged or necrotic cells, and active secretion from activated innate immune cells (such as monocytes, macrophages, and microglia) (15). We speculated that both mechanisms might contribute to the elevated peripheral HMGB1 levels in FES patients. HMGB1 can cause BBB breakdown and aggravate neuroinflammation and oxidative stress in neurological diseases (13–16). Additionally, it is worth mentioning that growing evidence (44–47), including our previous study (31), demonstrates blunted cytokine response to immune stimulations *in vitro*, implicating the state of immune tolerance or senescence in SCZ. Indeed, endotoxin and extracellular HMGB1 play a critical role in immune tolerance (6, 48, 49), which is not conducive to pathogen clearance and ultimately result in persistent low-grade systemic inflammation and in turn cause further detrimental injury to the intestinal mucosal barrier and tissue cells, thereby forming a vicious cycle.

Our result also showed a significant increase in C4 plasma levels in FES patients relative to HCs, which is consistent with a recent study conducted in patients with FEP (27). Genetic and animal studies have provided convincing evidence for the relationship between C4 and risk of developing SCZ via perturbation of synaptic remodeling (23, 25). So far, serological studies on complement levels in SCZ have remained inconsistent according to a previous systematic review and meta-analysis (50). Nevertheless, more recent individual studies still reported significant increases of C4 in SCZ (51–53). The causes for these discrepancies may relate to ethnicity, disease stage, heterogeneity of SCZ, and the impact of antipsychotics.

Additionally, our ROC analysis further revealed that endotoxin, HMGB1, and C4 concentrations in peripheral blood singly had moderate diagnostic effectiveness with AUC values ranging from 0.741 (C4) to 0.862 (HMGB1), while a combination of the three parameters could improve the diagnostic efficacy to 0.933. As is well established, an AUC value > 0.9 may imply a highly accurate diagnostic performance in clinical practice, suggesting that the three innate immune-related biomarkers selected in our study may be a promising combination for distinguishing FES from HCs.

In the present study, 20 patients were followed up prospectively for their clinical outcome at 8 weeks. We found the concentration of plasma HMGB1 in FES patients was significantly decreased after antipsychotic medication but still higher than HCs, whereas endotoxin and C4 were not affected by the treatment. Coupled with aforementioned cross-sectional results on the peripheral levels of the three biomarkers in SCZ patients, our finding indicates that they may be trait markers in SCZ. To date, only two studies have investigated their changes after treatment in SCZ patients, involving HMGB1 and C4, respectively (21, 53). Interestingly, these two studies were also about Chinese subjects. Consistent with our findings, Zhu et al. also showed a decrease of HMGB1 after risperidone treatment for 6 months, yet higher than HCs (21). However, Su et al. revealed that plasma C4 level could be suppressed by 4-week treatment with antipsychotics, becoming comparable to those of HCs (53). We speculate that the different disease duration and the length of follow-up might be, in part, responsible for this discordance. On the other hand, our results on the absence of correlations of endotoxin, HMGB1, and C4 levels with clinical efficacy as assessed by PANSS were generally similar to those observed in these two studies. Nevertheless, a prospective study enrolling 25 patients with FEP showed that higher baseline C4 level predicted worse clinical outcome at 1-year follow-up (54). In this study, clinical outcome was evaluated by WHO Personal and Psychiatric History Schedule, the focus of which lies on assessing disease relapse, whether or not accompanied with personality changes (54). It is worth noting that all the follow-up sample sizes in our and the other three studies were small, hence necessitating further research to draw a solid conclusion.

Several limitations should be mentioned in our study. Firstly, due to the small sample size, we did not consolidate our findings with another validation dataset. Therefore, caution should be taken in interpreting our results. Secondly, the present study could not establish the causal associations between increased endotoxin, HMGB1, C4, and SCZ. Moreover, the FES patients in our study had short-term psychotropic drugs exposure at baseline, hence, we cannot completely rule out the possible influences of this confounder on the plasma levels of the three biomarkers. Thirdly, we only examined total C4 levels rather than two isoforms of C4 including C4a and C4b, which are two functionally distinct isotypes (55). It is noted that evidence points to a more specific association of C4a in the peripheral blood and brain with SCZ risk (22, 23, 56, 57). Fourthly, as mentioned previously, we cannot draw definitive conclusions regarding whether endotoxin, HMGB1, and C4 could be affected by antipsychotic medication or remission status of disease, and thus these need to be further confirmed in longitudinal studies with larger sample size, longer follow-up periods and more comprehensive assessment of clinical outcomes. Finally, due to the naturalistic follow-up design, we did not stipulate the type and dosage of antipsychotics, notwithstanding different drugs might have different impacts on the three biomarkers, which should be clarified in the future.

## Conclusion

Despite these limitations, this study is the first to holistically explore endotoxin, HMGB1, and C4 in FES individuals with minimal exposure to antipsychotics, revealing that a combination of the three biomarkers in the peripheral blood can be used to distinguish FES patients from HCs, indicating its clinical usefulness. In addition, we found that endotoxin showed a significant positive correlation with auditory hallucinations, suggesting that endotoxin might be implicated in the pathogenesis of this important psychotic symptom, and might also be a potentially interesting target for future therapy. Future study should determine whether the immune variables measured here are specific to SCZ, in other words, whether they have the ability of differential diagnosis among various mental disorders.

## Data availability statement

The raw data supporting the conclusions of this article will be made available by the authors, without undue reservation.

## Ethics statement

The studies involving human participants were reviewed and approved by the Medical Ethical Committee of Beijing HuiLongGuan Hospital. The patients/participants provided their written informed consent to participate in this study.

## Author contributions

LT and YT initiated and directed the study. SC, MG, WC, and MX were responsible of recruitment of study subjects and clinical assessments. LT, YT, and SC performed statistical analysis and interpreted results. SC and LT wrote the first draft of the manuscript in consultation with YT. HF invited in evolving the ideas, analyzing data, and editing the manuscript. All authors read and approved the final manuscript.
